# Biomarkers of male hypogonadism in childhood and adolescence

**DOI:** 10.1515/almed-2020-0024

**Published:** 2020-04-21

**Authors:** Rodolfo A. Rey

**Affiliations:** Centro de Investigaciones Endocrinológicas “Dr. César Bergadá” (CEDIE), CONICET-FEI- División de Endocrinología, Hospital de Niños Ricardo Gutiérrez, Gallo 1330, C1425EFD, Buenos Aires, Argentina; Departamento de Histología, Biología Celular, Embriología y Genética, Facultad de Medicina, Universidad de Buenos Aires, C1121ABG, Buenos Aires, Argentina

**Keywords:** ambiguous genitalia, cryptorchidism, gonadal dysgenesis, hypergonadotropic hypogonadism, hypogonadotropic hypogonadism, micropenis, sexual development disorders, testicle

## Abstract

**Objectives:**

The objective of this review was to characterize the use of biomarkers of male hypogonadism in childhood and adolescence.

**Contents:**

The hypothalamic-pituitary-gonadal (HPG) axis is active during fetal life and over the first months of postnatal life. The pituitary gland secretes follicle stimulating hormone (FSH) and luteinizing hormone (LH), whereas the testes induce Leydig cells to produce testosterone and insulin-like factor 3 (INSL), and drive Sertoli cells to secrete anti-Müllerian hormone (AMH) and inhibin B. During childhood, serum levels of gonadotropins, testosterone and insulin-like 3 (INSL3) decline to undetectable levels, whereas levels of AMH and inhibin B remain high. During puberty, the production of gonadotropins, testosterone, and INSL3 is reactivated, inhibin B increases, and AMH decreases as a sign of Sertoli cell maturation.

**Summary and outlook:**

Based on our knowledge of the developmental physiology of the HPG axis, these biomarkers can be used in clinical practice to interpret the physiopathology of hypogonadism. Additionally, these markers can have diagnostic value in different forms of hypogonadism that may appear during childhood and adolescence.

## Introduction

Hypogonadism in males is typically defined as a testicular failure characterized by androgen deficiency. Although this definition is widely accepted in the endocrinology of adults, it is hardly useful in pediatric patients [1]. To better understand the difficulties that may arise from an inadequate use of this definition of hypogonadism in children and adolescents, it is necessary to consider the developmental physiopathology of the hypothalamic-pituitary-gonadal (HPG) axis.

## Developmental physiology of the HPG axis

**Figure 1:**
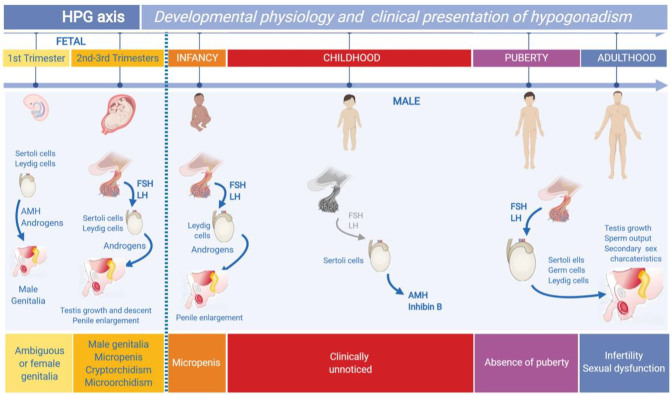
Ontogeny of the hypothalamic-pituitary-gonadal axis (HPG) in males and impact on the clinical presentation of hypogonadism. Gonad differentiation occurs during the first trimester of fetal life separately from pituitary gonadotropins. Testicular androgens and the anti-Müllerian (AMH) hormone induce male genital differentiation, and its absence results in female development. Hypogonadism in this period of life induces the development of ambiguous genitalia or female genitalia in XY individuals. In the second and third trimester, androgens induce testicular descent and penile growth. Primary and secondary hypogonadism result in micropenis, microorchidism, and/or cryptorchidism in newborns with male genitalia. During the first days of postnatal life, gonadotropin and androgen secretion is activated. Hypogonadism prevents penile growth. During childhood, gonadotropins and testosterone are generally low or even undetectable. Hypogonadism established in this period is not associated with evident clinical signs and can only be detected by AMH or inhibin B determination. At puberty, the HPG axis reactivates, thereby driving the typical development of secondary sexual characteristics. Hypogonadism can inhibit puberty totally or partially or cause infertility and sexual dysfunction in later stages of life. This Figure was modified using BioRender ({ut1}https://biorender.com/) under the authorization of [1]. © 2019 Elsevier Ltd.

Testis differentiation occurs by the 6th week of embryonic development (week 8 after last menstrual period (LMP)) before HPG axis function is activated [2]. The seminiferous cords originate from interaction of Sertoli cells, which surround germ cells, whereas Leydig cells appear in interstitial tissue. Sertoli cells secrete Anti-Müllerian hormones (AMH), which cause the regression of paramesonephric ducts or Müllerian ducts (primitive uterus and Fallopian tubes) during the 8th and 9th week of intrauterine life (Figure 1). At this stage, AMH is independent from pituitary gonadotropins, albeit from the second half of gestation it is sensitive to follicle stimulating hormone (FSH) [3]. Sertoli cells also secrete inhibin B, which is stimulated by FSH and controls negative feedback effect on pituitary production of FSH [4, 5]. Leydig cells produce androgens (Figure 1), which cause mesonephric ducts (Wolffian ducts) to develop into epididymis, vas deferens, and seminal vesicles. In addition, androgens induce the differentiation of the urogenital sinus and formation of external genitalia [6]. The synthesis of androgens is activated by human chorionic gonadotropin (hCG) action during the first trimester of gestation, and of pituitary luteinizing hormone (LH) in a later stage. Leydig cells also secrete insulin-like 3 factor (INSL3) which is in conjunction with androgens and induce the descent of the testis into the scrotal sac [7, 8].

Concentrations of all gonadal axis hormones in blood are low at birth and increase progressively from the first week of life [9]. Levels of gonadotropins, testosterone, and INSL3 are similar to those of adults until the sixth month of life, when they start to decline to low or undetectable levels [10]. High AMH levels persist in childhood, which is indicative of Sertoli cell immaturity [11, 12], whereas inhibin B partially decreases but remains detectable [5]. During childhood, the testicles grow in an unnoticeable wayloffset 4.5mm in clinical terms, being Sertoli cell population the one that most contributes to testes volume [13]. Despite the exposure to high androgen concentrations until 6 months of life, Sertoli cell maturation does not start, as they do not express androgen receptor [14–16]. After the first year of life, androgen receptor expression appears. However, Sertoli cells remain immature in childhood owing to low testosterone levels [17]. Germ cells only proliferate by mitosis but do not enter meiosis, which prevents spermatogenesis.

Pubertal onset is characterized by a reactivation of the gonadotrope, which starts the cyclical production of FSH and LH. FSH induces the proliferation of immature Sertoli cells. Thus, testis volume starts to increase progressively. LH induces Leydig cell secretion of testosterone. Increased levels of intratesticular testosterone induce the maturation of Sertoli cells, which refrain AMH production and stimulate inhibin B secretion [13]. Another characteristic of mature Sertoli cells is their ability to develop the blood-testis barrier (BTB) and functionally sustain adult spermatogenesis [18]. The significant proliferation of germ cells causes a remarkable increase of testes volume.

## Biomarkers of the HPG axis

### Pituitary hormones: LH and FSH

Gonadotropins LH and FSH share the alpha subunit with pituitary thyrotropin (TSH) and hCG, and owe their specificity to their beta subunit. LH and FSH are secreted by the pituitary gonadotrope in response to the stimulus of gonadotropin-releasing hormone (GnRH) produced by the hypothalamus.

#### Luteinizing hormone

LH binds to *luteinizing hormone* choriogonadotropin receptor (LHCGR), which is stimulated by LH and hCG and is present in Leydig cell membranes. LH stimulates testicular steroidogenesis resulting in an increase in circulating testosterone concentrations. LH has a trophic effect on Leydig cells, thereby inducing their proliferation (hyperplasia) and stimulating INSL3 secretion [19, 20]. Decreased levels of LH induce Leydig cell dedifferentiation into mesenchymal precursors and a reduction of androgen and INSL3 levels after the 3 to 6 month period of postnatal activation, which is typically known as "mini-puberty" [10, 13, 21]. During puberty, which generally begins at any point from the ages of 9 to 14 [22], LH stimulates the proliferation of Leydig cells and the production of androgens and INSL3.

**Figure 2:**
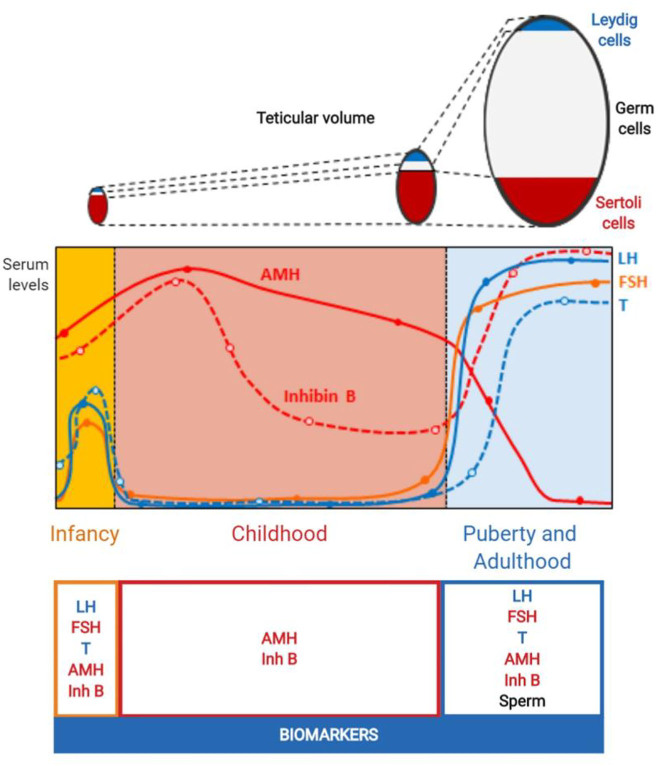
Ontogeny of the evolution of testicular volume from birth to adulthood. Seminiferous cords (Sertoli cells + germ cells) are the main component of the testes. From birth and during the prepubertal period (i. e., until 9–14, Tanner stage 1), the volume of seminiferous cords is determined by Sertoli cells, whereas germ cell proliferation determines testicular volume during puberty (i. e., Tanner stages 2 to 5) (adult spermatogenesis). This Figure was modified using BioRender (https://biorender.com/) under the authorization of [1]. © 2019 Elsevier Ltd.

Circulating LH levels are very low during the first postnatal hours [23] and increase during the first week of life [9] to remain at similar levels to those of puberty until 3 to 6 months of life [10]. Then, serum LH declines to non-detectable levels by a series of well-known mechanisms and remains stable until pubertal onset (Figure 2). During puberty, LH is secreted in a pulsatile fashion at 90 min intervals first during the night and later the whole day [24]. Circulating LH levels increase progressively during puberty following Tanner stages [12]. As it occurs with other gonadal axis hormones, LH levels must be determined based on Tanner stage [25] rather than age. This is due to considerable inter-individual variability in the age of pubertal onset and end in the general population [22].

#### Follicle stimulating hormone

FSH binds to its specific receptor follicle stimulating hormone receptor (FSHR), which is expressed in Sertoli cell membranes. FSH stimulates immature Sertoli cell proliferation (from fetal life until the onset of puberty) and determines testes size in this stage of life. FSH stimulates the secretion of AMH [26] and inhibin B [27]. As LH, serum FSH is low at birth [23] and progressively increases during the first week of life [9]. In males, FSH concentrations are slightly lower than those of LH in "mini-puberty" [12]. During childhood, FSH also declines, but not as steeply as LH (Figure 2). As a result, circulating FSH levels in this stage of life exceed those of LH [12, 28].

### Testicular hormones

Testicles contain two cell populations with endocrine function: Sertoli cells and Leydig cells. Endocrine activity of Leydig cells is clinically informative during postnatal activation or "mini-puberty" and puberty, whereas Sertoli cell activity is more informative in childhood.

#### Testosterone

Circulating levels of testosterone and LH undergo the same variations (Figure 2) i. e., they are low at birth [23] and progressively increase during the first month of life [9]. Although it is recommended that determination of steroid levels be performed by immunoassay [29], other steroids may be unspecifically detected during the first two and three weeks of life; therefore, extraction is required prior to determination to avoid overestimation [9]. From 3 to 6 months of life, circulating testosterone in plasma decreases to undetectable levels, and increases in Tanner stage 2 or 3 [12]. During childhood, estimation of the functional activity of Leydig cells can be based on circulating testosterone levels after hCG stimulation (2 to 3 IM injections of 1,500 to 2,500 IU at 48 h intervals [30].

Insulin-like 3 or Insulin-like 3 factorTesticular secretion of INSL3 is similar to that of testosterone [10, 13, 21]. However, INSL3 only reflects the long-term trophic effect of gonadotropins on Leydig cells, and INSL3 determination is not informative after acute hCG stimulation [19].

Anti-Müllerian hormoneAMH is a distinctive marker of prepuberal Sertoli cell population (Figure 2). Serum AMH decreases at birth and increases during the first weeks of life [9] to peak at 2–3 years of age, being 100-fold higher in males [11, 12, 31]. This is of clinical relevance, as samples from males require dilution for AMH concentrations to be within the range of detection of the immunoassays currently used in clinical laboratories.

Although basal AMH production is not dependent on gonadotropins [32], FSH stimulates testicular AMH secretion [26, 33–35]. In turn, increased testosterone concentrations inhibit AMH secretion [18, 36]. However, the increase of circulating androgen levels induced by medication is not enough to inhibit AMH [37]. Similarly, in males younger than 1 year, testosterone does not inhibit AMH production, as Sertoli cells do not express the androgen receptor in this stage of life [14, 38].

#### Inhibin B

Inhibins are dimeric proteins secreted by the gonads [39] with two isoforms with the same alpha subunit but distinct beta subunits. Inhibin B, which is complexed with a beta-B subunit, is the only form of inhibin with physiological relevance in males [40, 41]. In men, inhibin B is secreted in high amounts by Sertoli cells [4] and its production is stimulated by FSH [27, 34]. At the same time, inhibin B is the main inhibitor of FSH secretion from the pituitary gland. Levels of FSH are very elevated in patients with a depressed secretion or loss of inhibin B as in anorchia. However, during childhood, FSH may not be elevated [28], which is indicative of the hypothalamic–pituitary latency (gonadotrope) during that period of life.

Levels of inhibin B increase during the first weeks of life [9] reaching adult concentrations at 2 years of life [42, 43]. From 3 years of age, levels of inhibin B decrease slightly, but remain detectable (Figure 2) and higher than in girls. At puberty, inhibin B levels rise to a peak at Tanner stage 2 or 3 [4, 43, 44]. Thereafter, levels of inhibin B reflect Sertoli cell activity and interaction with germ cells.

## Male hypogonadism

Male hypogonadism in adults has been defined as [1] a testicular dysfunction reflected in androgen deficiency with or without impaired sperm production [45]. Based on the developmental physiology described above, all male infants and children would meet the criteria for hypogonadism, as they do not produce testosterone or sperm. However, Sertoli cells are active during childhood, thereby inducing a slight testicular growth (Figure 2) and the production of AMH [46] and inhibin B [42, 43]. Determination of Sertoli cells is useful as an indicator of testicular function in the pediatric population. For a definition of male hypogonadism to be applicable to children, diagnosis of a diminished testicular function should be established taking the testicular function expected for the age of the patient as a reference, which may involve Sertoli cells (AMH, inhibin B), Leydig cells (testosterone, INSL3), and/or germ-cells [47].

Considering this principle, male hypogonadism should not only be classified based on the constituent of the HPG axis primarily affected, but also on the period of life and the testicular population primarily affected (Tables 1 and 2).

**Table 1:** Fetal-onset male hypogonadism.

		Genitalia	Childhood					Puberty-Adulthood					
			LH	FSH	T	AMH	Inh B	LH	FSH	T	AMH	Inh B	Sperm.
Primary hypogonadism (Testicular)	Generalized gonadal failure												
	Gonadal dysgenesis	Female or ambiguous	N-H	N-H	L-ND	L-ND	L-ND	H	H	L-ND	L-ND	L-ND	Azoosp.
	Testicular regression syndrome Testicular torsion	Micropenis Empty scrotum	N-H	N-H	L-ND	L-ND	L-ND	H	H	L-ND	L-ND	L-ND	Azoosp.
	Klinefelter syndrome, XX Male	Male	N	N	N	N	N	H	H	N-L	L-ND	L-ND	Azoosp.
	Dissociated gonadal failure												
	Leydig cells												
	Hypoplasia/aplasia Steroidogenic defects	Female or hypovirilized	N-H	N	L-ND	N-H	N	H	H	L-ND	N-H	L-ND	Azoosp.
	INSL3 mutations	Cryptorchidism	N	N	N	N	N	N	N-H	N		N-L	Oligosp.
	Sertoli cells												
	FSH-R mutations	Small testicles	N	N	N	L	L	N	H	N	L	L	Oligosp.
	AMH mutations	PMDS	N	N	N	ND	N	N	N	N	ND	N	N
Secondary hypogonadism (Central)	Generalized gonadal failure												
	Multiple pituitary hormone deficiency	Micropenis, cryptorchidism	L	L	L	L	L	L	L	L	L	L	Oligosp./azoosp.
	Isolated central hypogonadism	Micropenis, cryptorchidism	L	L	L	L	L	L	L	L	L	L	Oligosp./azoosp.
	Dissociated gonadal failure												
	Multiple pituitary hormone deficiency	Small testicles	N	L	N	L	L	N	L	N		L	
	Isolated central hypogonadism: *TAC3 or TACR3* mutations	Micropenis, cryptorchidism	L	N	L	N	N	L	N	L		L	Oligosp./azoosp.
	*LHβ* mutations	Micropenis, cryptorchidism	L	N	L	N	N	L	H	L	H		Oligosp./azoosp.
	*FSHβ* mutations	Small testicles	N	L	N			H	L	N			Oligosp./azoosp.
Dual hypogonadism (Combined)	Generalized gonadal failure												
	Prader-Willi syndrome X-linked congenital adrenal hypoplasia	Micropenis, cryptorchidism	L-N	L-N	L-N	L	L	N	N	L	L	L	Oligosp./azoosp.

L, N, H: Low, Normal, High with respect to reference range for age in males ND: non-detectable. Male hypogonadism: an extended classification based on a developmental, endocrine physiology-based approach. Andrology. 2013;1(1):3–16. © 2012 American Society of Andrology and European Academy of Andrology.

**Table 2:** Postnatal-onset male hypogonadism.

		Childhood					Puberty-Adulthood					
		LH	FSH	T	AMH	Inh B	LH	FSH	T	AMH	Inh B	Spermatogenesis
Primary hypogonadism (Testicular)	Generalized gonadal failure											
	Orchitis Testicular torsion or trauma	N	.	L-ND	L-ND	L-ND	L	L	L-ND	L-ND	L-ND	Oligosp./azoosp.
	Down's syndrome	N-H	N-H	L-N	L-N	L-N	L	L	L	L	L	Azoosp.
	Varicocele	N	N	N	N	N	N-H	N	L-N	N	N	Teratozoosp./asthenozoosp.
	Chronic diseases: *Granulomatous disease, amyloidosis, cystic fibrosis, kidney failure*						L	L	L-ND	L-ND	L-ND	Oligosp./azoosp.
	Late-onset hypogonadism	Not applicable					N-H	N-H	L		L	
	Dissociated gonadal failure											
	Chromosome Y deletions: *AZF* Genetic mutations: *CILD1, USP9Y*	N	N	N	N	N	N	A	N		L	Oligosp./azoosp.
	Chemotherapy Abdomino-pelvic radiotherapy	N	N	N-L		N-L	N-H	H	N-L		L	Oligosp./azoosp.
	Drug therapies: *Spironolactone, ketoconazole*	N	N	N-L			N-H	N-H	L			Oligosp.
Secondary hypogonadism (Central)	Generalized gonadal failure											
	Pituitary and CNS damage: Tumors, histiocytosis, trauma, etc.	L	L	L	L	L	L	L	L	L	L	Oligosp./azoosp.
	Functional central hypogonadism: Chronic diseases, drug/alcohol abuse, etc.,						L-N	L-N	L		L	Oligosp./azoosp.
Dual hypogonadism (Combined)	Generalized gonadal failure											
	Brain radiotherapy + chemotherapy Lead poisoning Marihuana use Total body irradiation	L-N	L-N	L-N	L	L	L-N	L-N	L	L	L	Oligosp./azoosp.

L, N, H: Low, Normal, High with respect to reference range for age in males ND: non-detectable. Male hypogonadism: an extended classification based on a developmental, endocrine physiology-based approach. Andrology. 2013;1(1):3–16. © 2012 American Society of Andrology and European Academy of Andrology.

### Primary (“Hypergonadotropic”), secondary (“Hypogonadotropic”) or dual hypogonadism

Hypogonadism may be caused by a primary defect in the hypothalamus or pituitary gland or an abnormality in the gonads. In rare cases, both, the hypothalamic–pituitary axis and the testes present a primary defect, which originates dual or combined hypogonadism [47].

#### Primary hypogonadism

In adult medicine, primary hypogonadism (testicular or peripheral) is known as "hypergonadotropic" [1, 45] and is characterized by a primary defect in the testis. Deficient inhibin B and testosterone production reduces the negative feedback effect on the HPG axis, which results in an increased production of gonadotropins. This phenomenon does not always occur resulting in LH and FSH dissociation. Some examples of primary hypogonadism include Klinefelter syndrome, Testicular regression syndrome (TRS), and orchitis, to name a few.

#### Secondary hypogonadism

Secondary hypogonadism (hypothalamus–pituitary or central) is known as "hypogonadotropic" in adult medicine [1, 45] and is characterized by a primary defect in the hypothalamus or the pituitary gland. Impaired production of LH and FSH prevents the normal development of Leydig cells and seminiferous cords (Sertoli cells and germ cells). The developmental physiology of the HPG axis makes the diagnosis of these conditions challenging. LH and FSH dissociation may also occur. Examples of secondary hypogonadism include Kallmann syndrome, multihormonal pituitary hormone deficiency, and pituitary hormone deficiency after central nervous system surgery, to name a few.

#### Dual hypogonadism

There are rare conditions where both, the HPG axis and the gonads present primary damage. In contrast with primary and secondary hypogonadism, impairment of all testicular cell populations is concomitant and not secondary. Dual hypogonadism conditions include Prader–Willi syndrome and gonadal failure in oncologic patients treated with chemotherapy and cranial radiotherapy, among others.

### Generalized or dissociated gonadal failure

#### Generalized hypogonadism

In these cases, all testicular cell populations exhibit primary damage, concentrations are decreased, and germ-cell production is impaired. Examples include testicular dysgenesis and Kallmann syndrome (isolated hypogonadotropic hypogonadism with hyposmia).

#### Dissociated hypogonadism

Dissociated hypogonadism is characterized by primary damage in a specific testicular cell population. In the short or long term, the production of other populations becomes impaired at different degrees. Some examples include Leydig cell hypoplasia secondary to LHCG-R mutations, FSH deficiency for FSH beta-subunit gene mutations involving Sertoli cells, and post-chemotherapy gonadal failure, which primarily damages germ cells.

### Hypogonadism of fetal, childhood, pubertal or adult onset

The clinical manifestations of hypogonadism are dependent on the period of life where failure occurs. Fetal hypogonadism established during the first trimester causes a disorder of sex development (DSD), which manifests in the form of ambiguous or female genitalia at birth [48]. Gonadal dysgenesis is an example of generalized fetal hypogonadism, whereas Leydig cell hypoplasia is a dissociated form. Central hypogonadism does not result in genital ambiguity, as Leydig cell function during the first trimester of gestation is not dependent on pituitary gonadotropins, but on placental hCG. Hypogonadism occurred from the second trimester of fetal life, be it testicular, central or dual, results in micropenis or cryptorchidism in males without genital ambiguity [49–51]. As the HPG axis remains active for the first 3–6 months of postnatal life [9, 10], this period represents a window of opportunity to establish a diagnosis of hypogonadism [49–51].

Hypogonadism diagnosed in childhood may remain unnoticed. This is due to the fact that HPG activity decreases during childhood. For the condition to be diagnosed, suspicion or active screening is required (i. e., by baseline AMH or inhibin B determination, or measuring testosterone levels in response to hCG-induced stimulation). Otherwise, diagnosis will be delayed until puberty [52].

At puberty, male hypogonadism is characterized by the absence or interruption of normal pubertal development [22, 25]. As a result of androgen deficiency, secondary sex characteristics do not appear, i. e., body proportions typically are eunuchoid (upper/lower body proportion <1, with a span exceeding 6 cm), deepening of the voice is compromised, bone maturation is delayed, and testicular volume does not increase, which indicates disturbed spermatogenesis.

Hypogonadism established in adulthood is characterized by decreased libido, impotence and oligozoospermia [45]. Men of an older age may develop a mild androgen deficiency known as late-onset hypogonadism [53], which has similar symptoms to those of hypogonadism in young men.

## Clinical utility of HPG axis biomarkers in childhood and adolescence

### From birth to 3–6 months of life

During this period, the HPG is active, and all hormones are informative.

#### Newborns with ambiguous or female genitalia

In newborns with ambiguous genitalia, the causes of a DSD must be investigated. In patients with a 46,XY karyotype, the cause may be gonadal dysgenesis or generalized fetal‐onset primary hypogonadism established in the first trimester of gestation. These patients generally exhibit very low levels of AMH, inhibin B, testosterone and INSL3, whereas gonadotropins are elevated [48, 51, 54]. Imaging studies demonstrate the presence of uterus and fallopian tubes due to AMH deficiency. When genital ambiguity co-occurs with the absence of Müllerian structures, the cause may be dissociated primary fetal hypogonadism with a specific failure of the Leydigian sector. The causes may be Leydig cell hypoplasia secondary to LHCG-R mutations or an abnormality in the proteins involved in testicular steroidogenesis [55]. Differential diagnosis from gonadal dysgenesis is based on the presence of low testosterone levels, high LH levels, and AMH levels within normal range for males [56]. Hypogonadism is excluded when testosterone and AMH concentrations are high. Then, the cause of DSD may be insensitivity to androgens secondary to androgen-receptor mutations, a deficient DHT production in peripheral tissues secondary to 5*α*-reductase mutations, or a non-endocrine cause [48, 54, 57]. In patients with sex chromosome anomalies (i. e., deletions of the short arm of the Y chromosome; 45,X/46,XY or other mosaicisms involving the presence of the Y-chromosome), the cause of the DSD is gonadal dysgenesis.

A rare form of DSD 46,XY is persistent Müllerian duct syndrome (PMDS), which is characterized by cryptorchidism and fully-developed male genitals. Gonadotropins and testosterone are within the normal range for males, whereas AMH is very low or undetectable when the cause is an *AMH* mutation, and normal in *AMHR2* mutations [58]. The first case corresponds to dissociated primary fetal hypogonadism specifically affecting Sertoli cells. The second case corresponds to peripheral resistance to AMH, with the absence of hypogonadism.

In newborns with karyotype 46,XX, genital ambiguity occurs as the result of excess suprarenal (i. e., congenital suprarenal hyperplasia) [59] or placental androgen production (aromatase deficiency) [60]. These patients have ovaries, and AMH and inhibin B concentrations are within the normal range for females [56]. Nevertheless, genital ambiguity may be secondary to testicular tissue development in the form of ovotestis or dysgenetic testis [61]. In the two first cases, AMH and testosterone are generally in an intermediate point between normal ranges for males and females, whereas gonadotropins can be elevated or even within normal range in the presence of functional ovarian tissue. Cases have been reported of males born with karyotype 46,XX and normal male genitalia. The detection of these cases is based on discordance with an eventual karyotype developed during gestation. These patients exhibit normal HPG axis hormone levels for males until puberty, as described below.

#### Newborns with micropenis, cryptorchidism, and/or micro-orchidism

Micropenis, cryptorchidism and/or micro-orchidism are signs of HPG axis failure. This type of fetal hypogonadism establishes from the second trimester of gestation after male genital differentiation has started. Co-occurrence of low levels of LH, FSH, testosterone, INSL3, AMH, and inhibin B is highly suggestive of central fetal hypogonadism (hypogonadotropin) affecting all HPG axis sectors [34, 51, 62–64]. However, these low levels may also be due to a generalized primary testicular failure from the second trimester of gestation. TRS is characterized by undetactable levels of testicular hormones with elevated gonadotropin concentrations [51, 65].

### From 6 months to pubertal age

In this period of life, gonadotropins, testosterone, and INSL3 are uninformative, whereas Sertoli cells are of greater clinical utility.

If the condition is congenital but diagnosis was delayed, identifying the cause of the problem may be challenging. Low levels of AMH and inhibin B are indicative of Sertoli cell deficiency. However, it is difficult to establish whether the disorder is secondary to a primary testicular failure or a HPG axis failure. Gonadotropins may normalize during childhood in patients with primary hypogonadism (dysgenetic DSD or DSD caused by Leydig cell dysfunction, TRS or anorchidism). Otherwise said, primary male hypogonadism is not always "hypergonadotropic" at prepubertal age [28]. These patients show normal testosterone levels (i. e., undetectable), unless an hCG stimulation test is performed to determine the presence of functional Leydig cells. Undetectable levels of AMH and inhibin B are confirmatory of anorchidism.

In children without a perinatal history of micropenis, the probability of fetal hypogonadism is lower. Finding is generally incidental and occurs during evaluation of cryptorchidism, torsion, testicular trauma or oncologic treatments that may affect gonadal function. Again, gonadotropins and basal testosterone have poor diagnostic value, as gonadotropins do not increase during childhood, which is the period in which gonadal damage occurs (from 6 months of life) [28]. In patients without palpable gonads, detectable levels of AMH [66] or inhibin B [67] guarantees the presence of ectopic gonads, and testosterone levels increase after a hCG stimulation test [66]. Low levels of AMH [52, 66, 68, 69] or inhibin B [67, 68] are indicative of an abnormal testicular function. Cases have been reported of children with monorchidism with normal AMH and inhibin B values [70]. Circulating levels of INSL3 are not of clinical utility in this age group [71].

### At pubertal age

The absence of signs of pubertal development is suggestive of androgen deficiency. Although this abnormality can be secondary to primary hypogonadism, a testicular failure rarely affects the Leydig population, thereby inhibiting androgen secretion completely. In primary hypogonadism, the structures most frequently affected is the tubular sector, which translates into a small testicular volume [72]. Examples of this condition include Klinefelter syndrome [73, 74], XX males [75] and patients receiving chemotherapy [69]. These patients generally exhibit normal circulating levels of testicular hormones and gonadotropins until Tanner stage 3 of pubertal development. Then, primary hypogonadism becomes "hypergonadotropic".

Most frequently, the absence of pubertal development can be due to an HPG axis failure in the form of congenital or acquired central hypogonadism, a delayed reactivation of the HPG axis, or simple delayed puberty [22]. Differential diagnosis is challenging. Once general causes such as acute or chronic systemic diseases have been excluded, circulating levels of HPG-axis hormones are not necessarily informative. Gonadotropin concentrations at prepubertal levels are not of utility in differential diagnosis of central hypogonadism and simple delayed puberty, and GnRH [76] (or analogs) stimulation tests are required [77]. The presence of other pituitary deficiencies facilitates diagnosis, as they are indicative of gonadotropin deficiency. Testosterone and INSL3 remain at prepubertal levels and are not useful to distinguish central hypogonadism from simple delayed puberty [78]. In contrast, diagnosis is confirmed by AMH and inhibin B levels, as they are lower in patients with central hypogonadism as compared to those with simple delayed puberty [78, 79].

As in primary hypogonadism, central hypogonadism can affect all cell populations (i. e., generalized) or initially affect a single sector of the HPG axis. Examples of dissociated central hypogonadism include tachykinin Precursor 3 (TAC3) and tachykinin receptor 3 (TACR3) mutations [80] and LH beta sub-unit mutations [81], which manifest in the form of low LH levels and normal FSH levels. Other examples are FSH beta subunit mutations [82], which are associated with a decline in FSH and LH production and normal androgen levels.

Dual hypogonadism is characterized by concomitant HPG axis and gonad dysfunction. This condition can be congenital of which hypogonadism is a late manifestation, as in the case of Prader–Willi syndrome [83, 84] and delayed-onset X-linked adrenal hypoplasia congenita due to dosage-sensitive sex reversal, adrenal hypoplasia critical region, on chromosome X, gene 1 (DAX-1) gene mutations [85]. Dual hypogonadism can also be acquired as in the case of patients exposed to chemotherapy, which primarily affects the testes, and cranial radiotherapy, which affects the hypothalamus. Although gonadal hormone levels are low, gonadotropins do not increase. In other words, these conditions mimic eugonadotropic hypogonadism.

## Conclusions

Hypogonadism may have a fetal or postnatal origin and has different clinical manifestations according to the period of life in which it is established. This condition may affect any functional testicular component, or initially involve a single component, thereby resulting in specific clinical and biochemical manifestations. Primary damage generally occurs to the gonads or the HPG axis and rarely affects the two systems concomitantly. Gonadotropins and androgens are useful biomarkers in newborns and pubertal males, whereas AMH and inhibin B are more informative in childhood.
